# Interventricular differences in sodium current and its potential role in Brugada syndrome

**DOI:** 10.14814/phy2.13787

**Published:** 2018-07-16

**Authors:** Kirstine Calloe, Gary L. Aistrup, José M. Di Diego, Robert J. Goodrow, Jacqueline A. Treat, Jonathan M. Cordeiro

**Affiliations:** ^1^ Department of Veterinary and Animal Sciences Section for Anatomy, Biochemistry and Physiology University of Copenhagen Frederiksberg Denmark; ^2^ Department of Experimental Cardiology Masonic Medical Research Laboratory Utica New York; ^3^ Lankenau Institute for Medical Research Wynnewood Pennsylvania

**Keywords:** Action Potentials, left ventricle, patch clamp, right ventricle, sodium current

## Abstract

Brugada syndrome (BrS) is an inherited disease associated with ST elevation in the right precordial leads, polymorphic ventricular tachycardia (PVT), and sudden cardiac death in adults. Mutations in the cardiac sodium channel account for a large fraction of BrS cases. BrS manifests in the right ventricle (RV), which led us to examine the biophysical and molecular properties of sodium channel in myocytes isolated from the left (LV) and right ventricle. Patch clamp was used to record sodium current (*I*_Na_) in single canine RV and LV epicardial (epi) and endocardial (endo) myocytes. Action potentials were recorded from multicellular preparations and single cells. mRNA and proteins were determined using quantitative RT‐PCR and Western blot. Although LV wedge preparations were thicker than RV wedges, transmural ECG recordings showed no difference in the width of the QRS complex or transmural conduction time. Action potential characteristics showed RV epi and endo had a lower *V*_max_ compared with LV epi and endo cells. Peak *I*_Na_ density was significantly lower in epi and endo RV cells compared with epi and endo LV cells. Recovery from inactivation of *I*_Na_ in RV cells was slightly faster and half maximal steady‐state inactivation was more positive. *β*2 and *β*4 mRNA was detected at very low levels in both ventricles, which was confirmed at the protein level. Our observations demonstrate that *V*_max_ and Na^+^ current are smaller in RV, presumably due to differential Na_v_1.5/*β* subunit expression. These results provide a potential mechanism for the right ventricular manifestation of BrS.

## Introduction

Brugada syndrome (BrS) is an inherited disease associated with ST elevation or a broad J‐wave in the right precordial leads, phase 2 reentry, polymorphic ventricular tachycardia (PVT), and sudden cardiac death in adults (Brugada and Brugada 1992). The ECG pattern of BrS is often concealed, but can be unmasked or modulated by several factors including a number of Na^+^ channel inhibitors (Rolf et al. 2003).

The mechanisms underlying the syndrome are still being debated. Originally, BrS was linked to sudden cardiac death in adults with minor or no structural abnormalities in the heart (Brugada and Brugada 1992). However, recent studies suggest a pathophysiological component in hearts from BrS patients such as increased fibrosis and fatty infiltration as well as decreased connexin proteins, particularly in the right ventricular outflow tract (RVOT) leading to right ventricular conduction slowing. This is the basis of the depolarization hypothesis, and it appears that BrS is associated with fibrosis, whether or not patients carry a *SCN5A* mutation (Nademanee et al. 2015). The repolarization hypothesis relies on an imbalance in depolarizing and repolarizing currents in the early parts of the cardiac action potential (AP). A decrease in inward sodium channel currents or an increased transient outward K^+^ currents (*I*_to_) during phase 1 will accentuate the spike‐and‐dome morphology of the action potential mainly in the right ventricle (RV) epicardium (epi) generating a transmural voltage gradient that leads to the characteristic ECG changes (Calloe et al. 2009; Cordeiro et al. 2009) (for review see Wilde et al. (2010)). Both hypothesis have clinical and experimental support and may not be mutually exclusive.

Several genes have been linked to the development of BrS (for review see Sarquella‐Brugada et al. (2016). The most prevalent gene linked to BrS is mutations in the *SCN5A* encoding the Na_v_1.5 channel *α*‐subunit accounting for at least 30% of the BrS cases (Nielsen et al. 2013). Other mutations linked to BrS include *SCN1B* encoding the sodium channel *β*1‐subunit (Watanabe et al. 2008), and the glycerol‐3‐phosphate dehydrogenase 1 gene (London et al. 2007), all of which lead to a reduction in peak sodium channel current (*I*_Na_). Besides mutations affecting the sodium current, mutations in calcium channel subunits resulting in a loss of function have been reported (Antzelevitch et al. 2007; Cordeiro et al. 2009) as well as mutations resulting a gain of function of I_to_ (Delpón et al. 2008; Giudicessi et al. 2011; Ohno et al. 2011).

Interestingly, despite the fact that mutations in *SCN5A* would affect all Na^+^ channels in the heart, often these mutations only manifest in a chamber‐specific manner. For example, several *SCN5A* mutations have been linked to atrial fibrillation with no ventricular manifestations noted in the patients (Darbar et al. 2008). Similar observations have been noted in the ventricles (i.e., BrS and Long QT syndrome [LQTS]) (3) with no atrial manifestations. Presumably, a loss‐of‐function mutation in Na_v_1.5 would affect all Na^+^ channels in the ventricle; yet, BrS typically manifests in only the RV. In addition, a flecainide challenge which would also inhibit *I*_Na_ in both chambers often unmasks BrS only in the right ventricle. Previous studies have shown that there are tissue‐specific differences in *I*_Na_ in the canine heart. It is well established that Purkinje fibers have a high *V*_max_ and the largest *I*_Na_ in the heart, as they are adapted for rapid conduction (Gilmour and Zipes 1980). We and others have found that atrial *I*_Na_ has a higher current density and a more negative steady‐state mid‐inactivation potential compared to ventricular *I*_Na_ (Li et al. 2002; Burashnikov et al. 2007; Calloe et al. 2011), resulting in a larger proportion of Na^+^ channels in atria residing in the inactivated state at normal resting membrane potentials (Calloe et al. 2011). In the left ventricle, epi *I*_Na_ has a significantly more negative steady‐state mid‐inactivation potential compared with endodardial (endo) (Cordeiro et al. 2008). Similar to atrial *I*_Na_, the more negative steady‐state mid‐inactivation potential will tend to leave a larger fraction of endo *I*_Na_ channels in an inactivated state (Li et al. 2002; Burashnikov et al. 2007).

We hypothesize that differential expression of *I*_Na_ and regional differences in *I*_Na_ kinetics may explain why BrS is predominantly affecting RV. In this study, we isolated canine ventricular myocytes from RV and LV regions of the heart and examined the biophysical and molecular differences in peak Na^+^ current. Results of our study showed that RV cells exhibited a lower peak Na^+^ current compared to LV cells; no dramatic difference on steady‐state activation or inactivation was observed. Molecular analysis revealed that RV tissue cells had a differential expression of the various subunits that comprise voltage‐gated Na^+^ channels consistent with the lower *I*_Na_ density. Our findings indicate that Na^+^ current is smaller in RV cells and may contribute to the right ventricular manifestation of BrS.

## Methods

Adult mongrel dogs of either sex (age 1–4 years) were used for all experiments and this investigation conforms to the Guide for Care and Use of Laboratory Animals published by the National Institutes of Health (The Eighth Edition of the *Guide for the Care and Use of Laboratory Animals* [NRC 2011]). All protocols were approved by the Institutional Animal Care and Use Committee. Dogs were anticoagulated with heparin (1000 U/kg. i.v.) and anesthetized with pentobarbital (30–35 mg/kg, i.v.). The chest was open via a left thoracotomy, the heart excised, and placed in cold cardioplegic solution of the following composition (in mmol/L): NaCl 129, KCl 12, NaH_2_PO_4_ 0.9, NaHCO_3_ 20, CaCl_2_ 1.8, MgSO_4_ 0.5, and glucose 5.5.

### Ventricular wedge preparation

Transmural RV or LV wedges of up to 3 × 2 cm were dissected and cannulated through a branch of the right coronary artery or the left anterior descending coronary artery, respectively. The preparations were initially arterially perfused with cold cardioplegic solution. Subsequently, the wedges were placed in a tissue bath and perfused with Tyrode's solution of the following composition (mmol/L): 129 NaCl, 4 KCl, 0.9 NaH_2_PO_4_, 20 NaHCO_3_, 1.8 CaCl_2_, 0.5 MgSO_4_, 5.5 glucose, and buffered with 95% O_2_ and 5% CO_2_ (37.0 ± 0.5°C). The perfusate was delivered at a constant pressure (45–50 mmHg). Transmural ECGs were recorded using two Ag/AgCl half cells placed at ∼1 cm from the epi surface (+) and endo surface (−) of the preparation. APs were simultaneously recorded from the epi and endo surface using floating microelectrodes filled with 2.7 mol/L KCl (10–30 MΩ). Pacing was delivered to the endo surface at twice diastolic threshold at a basic cycle length (BCL) = 1 sec. Recordings were obtained using a high‐input‐impedance amplification system (Electro 705 Electrometer, World Precision Instruments). The signals were digitized at 20 kHz (model 1401 AD/DA system, Cambridge Electronic Designs [C.E.D.]) using Spike 2 acquisition software (C.E.D.). All amplified signals were analyzed using Spike 2 for Windows (Cambridge Electronic Design [CED], Cambridge, UK).

### Isolated tissue action potential recordings

Right ventricle or left ventricle epi slices (~2 mm thick) were isolated using a dermatome (Davol, Cranston, RI) and superfused with Tyrode's solution. All preparations were allowed to equilibrate until the action potentials reached steady state. Tissues were stimulated at basic cycle lengths (BCL) of 500–2000 msec (2–5 msec pulse duration) delivered through Teflon‐coated silver bipolar electrodes. Transmembrane action potentials were recorded from tissue using glass microelectrodes filled with 2.7 mol/L KCl. Electronic equipment similar to the one described in the Ventricular Wedge Preparation section was used.

### Isolation of ventricular cells

Left ventricle or right ventricle cells were prepared as previously described with minor modifications (Di Diego et al. 2002; Calloe et al. 2010). Left ventricular or right ventricular wedge preparations were dissected out and initially perfused with nominally Ca^2+^‐free solution (mmol/L): NaCl 129, KCl 5.4, MgSO_4_ 2.0, NaH_2_PO_4_ 0.9, glucose 5.5, NaHCO_3_ 20, and bubbled with 95% O_2_/5% CO_2_ containing 0.1% BSA for a period of about 5 min. The preparation was then subjected to enzyme digestion with the nominally Ca^2+^‐free solution supplemented with 0.5 mg/mL collagenase (Type II, Worthington), 0.1 mg/mL protease (Type XIV, Sigma), and 1 mg/mL BSA for 8–12 min. For isolation of ventricular cells, thin slices of tissue (approximately 2 mm thick) from the epi and endo were shaved from the wedge using a dermatome (Davol, Cranston, RI). The tissue slices were then placed in separate beakers, minced, and incubated in fresh buffer containing 0.5 mg/mL collagenase, 1 mg/mL BSA, and agitated. The supernatant was filtered, centrifuged at 200 rpm for 2 min, and the myocyte‐containing pellet was stored in 0.5 mmol/L Ca^2+^ HEPES buffer at room temperature.

### AP recordings from single myocytes

APs from ventricular cells were recorded using whole cell patch pipettes coupled to a MultiClamp 700A amplifier (Axon Instruments, Foster City, CA) as previously described (Murphy et al. 2011; Cordeiro et al. 2012; Calloe et al. 2016). Briefly, cells were superfused with HEPES buffer of the following composition (mmol/L): 126 NaCl, 5.4 KCl, 1.0 MgCl_2_, 1.8 CaCl_2_, 10 HEPES, and 11 glucose. pH adjusted to 7.4 with NaOH. The patch pipette solution had the following composition (in mmol/L): 90 K‐aspartate, 45 KCl, 1.0 MgCl_2_, 5 EGTA, 5 MgATP, 5 HEPES, and 10 NaCl (pH 7.2 with KOH). The resistance of the electrodes was 2–4 MΩ when filled with the pipette solution. APs were elicited using a 3‐msec current pulse at 120% threshold amplitude, and cells were paced at cycle lengths of 0.5 and 1 Hz. APs were acquired at 50 kHz and filtered at 5 kHz.

### Voltage clamp recordings of peak *I*_Na_

Early sodium current, *I*_Na_, was measured as previously described with minor modifications (Cordeiro et al. 2008; Calloe et al. 2011). Experiments were performed using a MultiClamp 700A (Molecular Devices, Foster City, CA). Command voltages were delivered and data acquired via a DigiData 1322 computer interface using pClamp 9 (Molecular Devices) with data stored on computer hard disk. Patch pipettes were pulled from borosilicate glass (1.5 mm o.d. and 1.1 mm i.d.) on a Model PP‐830 vertical puller (Narashige Instruments, Japan). The electrode resistance was 0.9–2.0 MΩ when filled with the internal solution (see below). The membrane was ruptured by applying negative pressure and series resistance compensated by 75–80%. Whole cell current data were acquired at 20–50 kHz and filtered at 5 kHz. Currents were normalized to cell capacitance.

External solution contained (in mmol/L): NaCl 15, Choline Cl 120, Na^+^ acetate 2.8, CaCl_2_ 0.5, KCl 4, MgCl_2_ 1.5, CoCl_2_ 1, glucose 10, HEPES 10, NaOH 5, BaCl_2_ 0.1, and pH adjusted to 7.4 with NaOH/HCl. The pipette solution contained (mmol/L): CsF 120, NaCl 15, MgCl_2_ 1, KCl 5, HEPES 10, Na_2_ATP 4, EGTA 10, and pH = 7.2 with CsOH. Peak sodium current was dramatically reduced in the low extracellular sodium to ensure adequate voltage control, as gauged by the slope of a Boltzmann fit to the steady‐state activation curve (Kaab et al. 1996). When measuring sodium channel kinetics and density the holding potential was −120 mV to recruit all available sodium channels. In addition, recordings of *I*_Na_ were made 5 min after rupture to minimize the effects of time‐dependent negative shift of steady‐state inactivation that occurs in conventional voltage clamp experiments. Whole cell currents were analyzed using Clampfit 9 (Molecular Devices).

### Quantitative real‐time PCR

qPCR analysis was performed with the QuantStudio 6 Flex Real‐Time PCR System (Applied Biosystems, CA). Total RNA was extracted with RNAeasy Micro (cells), Trizol, and/or Mini Kits (tissues) (Qiagen, CA). One thousand nanogram total RNA from each of the pooled PVC cells/tissues or atrial tissue samples was reverse transcribed with SuperScriptTM First Strand Synthesis System for RT‐PCR (Invitrogen, CA). Real‐time PCR was performed in triplicates for every sample using primers listed in Table 1. Using SYBR Green/ROX probe (Thermo Fisher, MA), averaged *C*_t_ values of each qPCR reaction from the target gene were normalized with the average *C*_t_ values of the housekeeping gene 18S, which ran in the same reaction plate to obtain the ∆*C*_t_ value (Barajas‐Martinez et al. 2017; Goodrow et al. 2017). Expression was normalized from ∆*C*_t_ values for each gene against reference housekeeping gene 18S, using the formula 2^−∆∆Ct^ (1 × 10^6^) (Livak and Schmittgen 2001):

**Table 1:** Oligonucleotide sequences of the primers used for RT‐PCR

Gene name	Forward primer	Reverse primer
SCN5A	CACCATGTGCATCGTCCTTAAC	CCATGAGGCTCAGGATGACAAT
SCN1B	TCTTCTTCGAGAACTACGAG	CATACATCATGATCTCCGAC
SCN2B	TACACAGTGAACCACAAAC	CAGGTTAATGATCTTCATGC
SCN3B	ATATTGCTACAGGAAGGTCTC	GCTCTCTTTGTTCTCTGA
SCN4B	AAATTCAGCTCATAGACGG	CTTTCTTTAGTGGAACCCTC
18S	CGCCGCTAGAGGTTGAAATTC	TCCGACTTTCGTTCTTGATTAATG

### Western blotting

Protein analysis was performed as previously described with minor modifications (Barajas‐Martinez et al. 2009; Calloe et al. 2010). Ventricular tissue from three dogs was snap frozen in liquid nitrogen and stored in −80°C prior to protein isolation. Membrane proteins were isolated using the Proteo Extract Native Membrane Kit (Calbiochem) according to the manufacturer's protocol. Protein concentration was determined by BCA assay (Pierce BCA Protein Assay). Samples were denatured 10 min at 65°C with 355 mmol/L *β*‐mercaptoethanol, separated on precast polyacrylamide 4–15% Tris‐HCl gels (BioRad) and transferred to polyvinylidene fluoride (PVDF) membranes. The PVDF membranes were incubated overnight at 4°C with the following primary antibodies. Rabbit polyclonal Anti‐Nav *β*1‐*β*4 (1:3000 dilution, Alomone Labs) and mouse monoclonal antitubulin (1:3000, Abcam) were used as a loading control. Secondary antibodies were HRP‐conjugated goat anti‐rabbit IgG (1:10000, BioRad) and goat anti‐mouse IgG (1:10000, Bio‐Rad).

### Statistics

Results are presented as Mean ± SEM. Statistical analysis was performed using an ANOVA test followed by a Student–Newman–Keuls test or a Student's *t*‐test, as appropriate, using SigmaStat software. A *P* < 0.05 was considered statistically significant.

## Results

**Figure 1:**
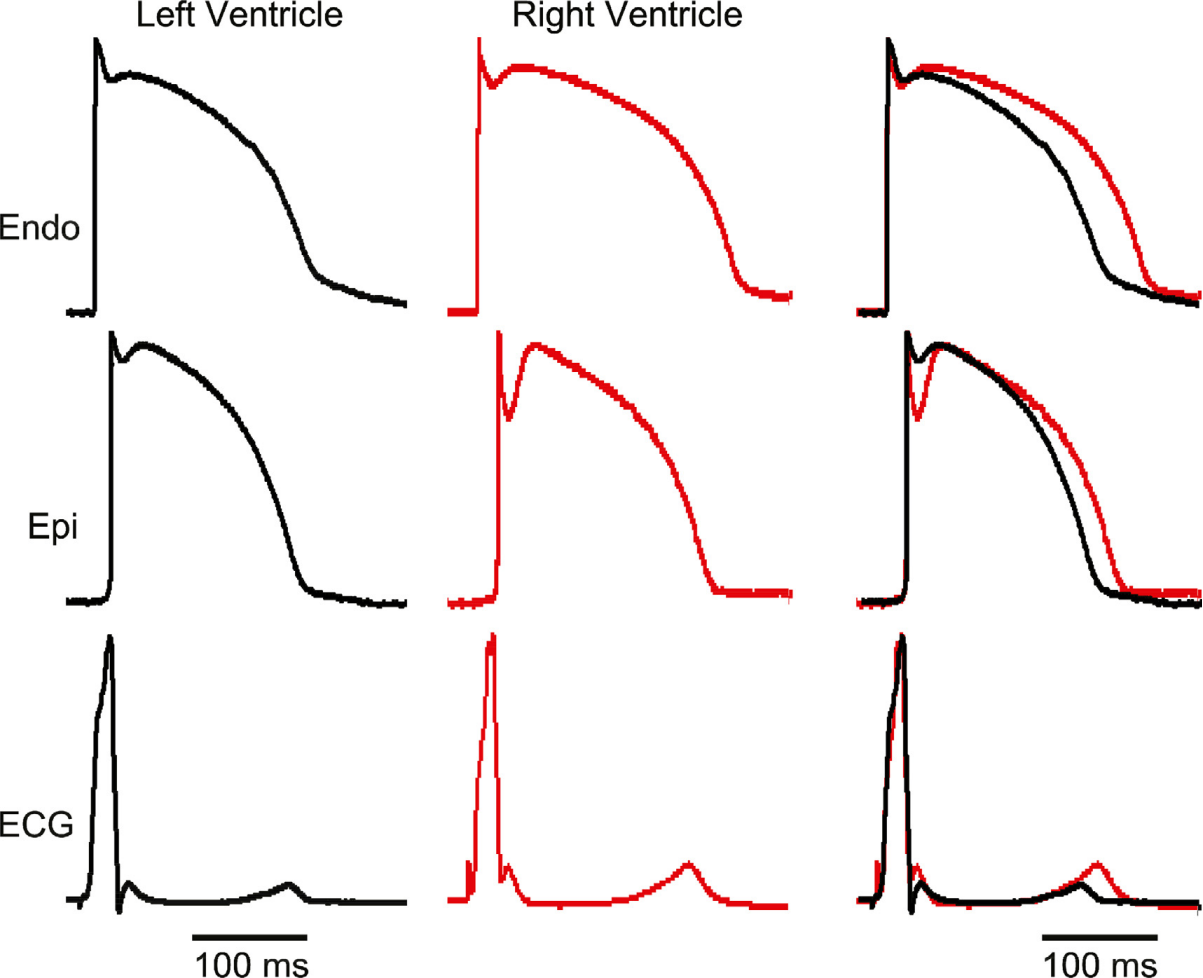
Representative recordings of endocardial (endo) and epicardial (epi) action potentials (APs) and the corresponding transmural‐ECG (ECG) obtained from a RV and LV preparation. Preparations were stimulated from the endo surface at a BCL = 1 sec. Although LV preparations were thicker than RV preparations, the superimposed ECGs show that transmural conduction time was similar.

As an initial basis of comparison, APs and the transmural ECG were simultaneously recorded from both the right and left ventricular wedge preparation. Figure 1 shows AP recordings from epi and endo layers (upper and middle traces) and the corresponding ECG (lower trace) from a right and left wedge preparation paced at BCL of 1000 msec. The epi recording from the RV wedge showed a much larger phase 1 repolarization compared to the LV wedge epi recording, as previously described (Calloe et al. 2010). The endo recording was similar in RV and LV preparations. Although the thickness of the LV wedge was always greater than the RV wedge, analysis of the transmural conduction time revealed no significant difference between RV and LV wedges (13.7 ± 2.0 msec vs. 15.0 ± 1.6 msec, respectively; *n *=* *8). Table 2 summarizes the electrophysiological parameters from the wedge experiments.

**Table 2:** Electrophysiological parameters from wedges

	LV Epi	RV Epi	LV Endo	RV Endo
APD50	135.2 ± 4.7 msec (*n* = 8)	147.8 ± 5.8 msec (*n* = 8)	165.8 ± 3.9 msec (*n* = 8)	162.3 ± 4.5 msec (*n* = 8)
APD90	165.8 ± 3.9 msec (*n* = 8)	181.5 ± 5.3 msec (*n* = 8)^a^	200.8 ± 2.5 msec (*n* = 8)	205.68 ± 3.0 msec (*n* = 8)
Notch, Ph1% of Ph0	15.4 ± 4.9% (*n* = 8)	22.5 ± 3.2% (*n* = 8)^a^	13.9 ± 1.7% (*n* = 8)	14.4 ± 2.6% (*n* = 8)
Notch, Ph1% of Ph0	13.2 ± 2.7% (*n* = 8)	21.0 ± 2.9% (*n* = 8)^a^	2.1 ± 2.1% (*n* = 8)	5.2 ± 2.1% (*n* = 8)

a Significantly different versus LV Epi (*P* < 0.05).

**Figure 2:**
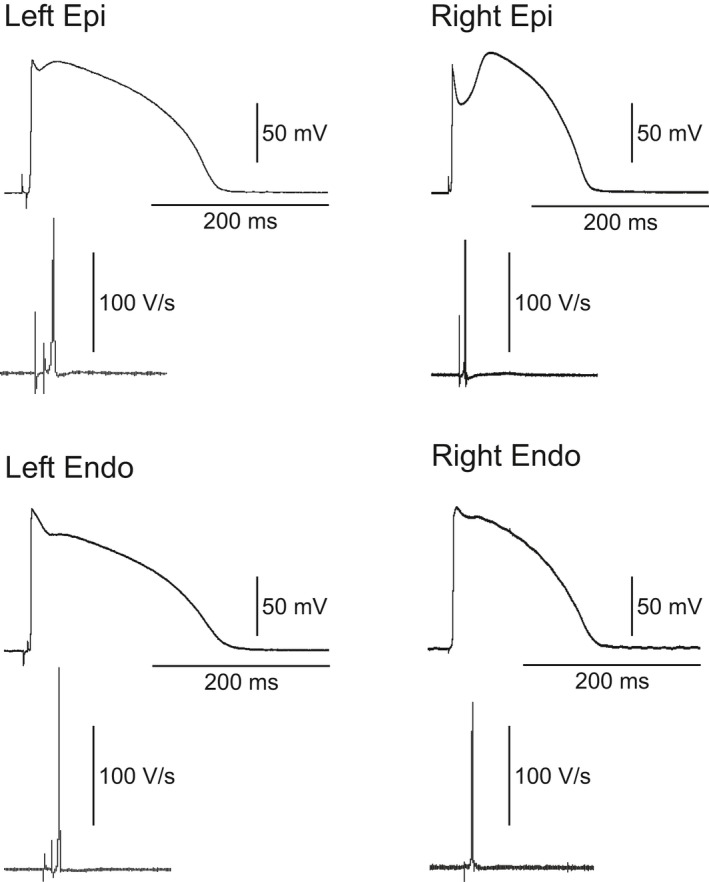
Representative action potentials and corresponding *V*_max_ recordings from RV and LV epi and endo slices. AP recordings were taken from preparations paced at a BCL = 1 sec using recordings high‐resistance microelectrodes.

The similarity in conduction velocity across the ventricular wall even though the thickness of the wedges was different suggests that (1) RV wedges have a lower upstroke velocity (an indirect assessment of Na^+^ channel current) and/or (2) RV wedges have a greater gap junctional resistance. As floating microelectrodes do not provide an accurate measure of upstroke velocity, we measured upstroke velocity in RV and LV epi and endo tissue slices. Thin sheets of epi or endo muscle were shaved from the right or left ventricle using a dermatome and APs were measured using high‐resistance microelectrodes (Fig. 2). Both RV and LV Epi slices exhibited a spike and dome morphology with RV tissue showing a greater phase 1 repolarization. Analysis of *V*_max_ showed that LV epi had a higher *V*_max_ (217.2 ± 15.2 V/sec, *n *=* *4) compared to RV epi (183.3 ± 14.3 V/sec, *n *=* *4, *P* < 0.05). Similarly, LV endo had a slightly higher *V*_max_ (216.2 ± 17.3 V/sec, *n *=* *4) compared to RV endo (198.1 ± 18.8 V/sec, *n *=* *4, *P *= N.S.) suggesting that LV tissue may have a greater *I*_Na_ density compared to RV tissue. Table 3 summarizes the electrophysiological parameters from the tissue slice experiments.

**Table 3:** Electrophysiological parameters from tissue slices

	LV Epi	RV Epi	LV Endo	RV Endo
APD50	145.8 ± 16.3 msec (*n* = 4)	138.7 ± 4.6 msec (*n* = 4)	150.7 ± 16.1 msec (*n* = 4)	125.7 ± 13.6 msec (*n* = 4)
APD90	194.9 ± 12.2 msec (*n* = 4)	163.8 ± 6.5 ms (*n* = 4)	222.4 ± 9.4 ms (*n* = 4)	195.1 ± 3.0 msec (*n* = 4)
AP amplitude	100.7 ± 4.5 mV (*n* = 4)	91.4 ± 1.7 mV (*n* = 4)	107.8 ± 4.0 mV (*n* = 4)	99.1 ± 2.5 mV (*n* = 4)
Resting membrane potential	−91.7 ± 1.6 mV (*n* = 4)	−87.3 ± 1.2 mV (*n* = 4)	−91.5 ± 1.8 mV (*n* = 4)	−90.2 ± 1.3 mV (*n* = 4)

We further examined *V*_max_ in single epi and endo cells isolated from the RV and LV (described in Methods). Similar to results obtained in tissue slices, analysis of *V*_max_ at the cellular level revealed that the LV had a higher *V*_max_ (353.4 ± 12.7 V/sec, *n *=* *9 for LV epi and 336.1 ± 15.6 V/s, *n *=* *8 for LV endo) than cells isolated from the RV (303.4 ± 15.4 V/sec, *n *=* *9 for RV epi and 288.1 ± 21.0 V/sec, *n *=* *9 for RV endo).

**Figure 3:**
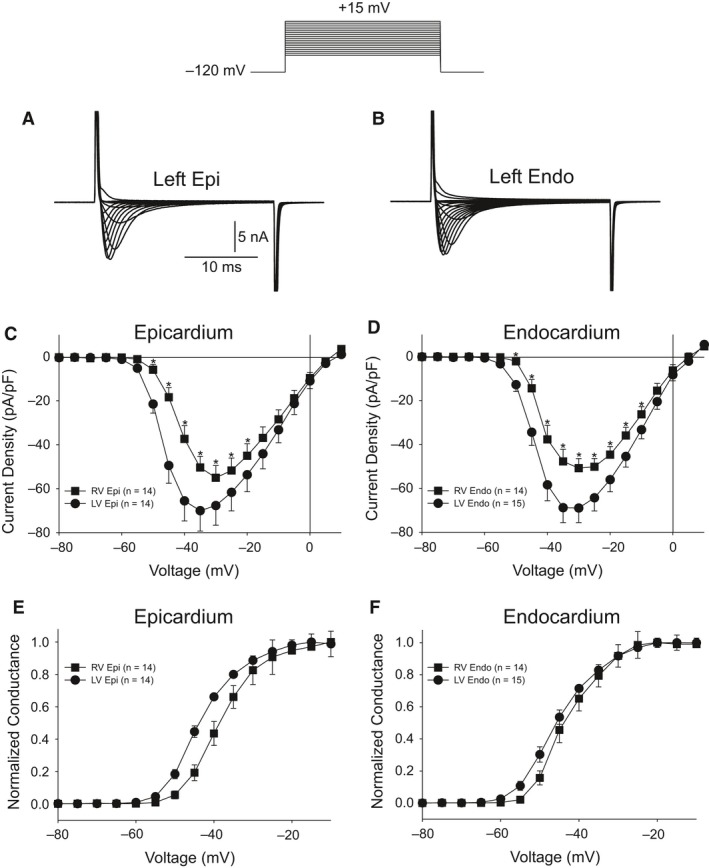
Representative *I*_Na_ recordings from a LV epi (A) and LV endo myocyte (B). Current recordings were obtained at test potentials between −80 and 15 mV in 5 mV increments. The holding potential was −120 mV. (C) I–V relation for RV (*n* = 14) and LV myocytes (*n* = 14) from the epicardium showing a small but significant reduction in *I*_Na_ magnitude in RV epi cells. (D) I–V relation for RV (*n* = 14) and LV myocytes (*n* = 15) from the endocardium showing a small but significant reduction in *I*_Na_ magnitude in RV endo cells. (E–F) Steady‐state activation relation for epi (E) and endo (F) cells. Data from the I–V curve were normalized and plotted against their test potential. *P < 0.05.

The differences in *V*_max_ observed between LV and RV may suggest that Na^+^ current in LV tissue may be larger. To test this hypothesis, we measured peak *I*_Na_ in epi and endo cells isolated from the RV and LV. Peak *I*_Na_ was measured in low extracellular sodium buffer and at room temperature to ensure adequate voltage control. Representative *I*_Na_ traces recorded from a LV epi and endo cell are shown (Fig. 3A and B). Analysis of the I–V relation (Fig. 3C) showed that peak *I*_Na_ density was larger in LV epi cells compared to RV epi cells (−70.0 ± 9.2 pA/pF vs. −55.1 ± 5.6 pA/pF, respectively, *P* < 0.05) but no shift in the activation threshold was noted. Similarly, *I*_Na_ density was larger in LV endo cells compared to RV endo cells (−68.9 ± 6.6 pA/pF vs. −50.8 ± 4.4 pA/pF, respectively, *P* < 0.05) (Fig. 3). We also measured steady‐state activation from the I–V curves. Chord conductance was determined using the ratio of current to the electromotive potential for the cells shown Panels C and D and a Boltzmann curve was fit to the data. Analysis of steady‐state activation showed mid‐activation voltages (*V*_1/2_) of −43.53 ± 0.42 mV, *k* = 5.03 ± 0.33 for LV epi and −38.51 ± 0.25 mV, *k* = 4.87 ± 0.21 for RV epi (Fig. 3E). Similarly, mid‐activation voltages (*V*_1/2_) of −45.43 ± 0.35 mV, *k* = 5.51 ± 0.32 for LV endo and −43.25 ± 0.45 mV, *k* = 4.83 ± 0.40 for RV endo (Fig. 3F).

**Figure 4:**
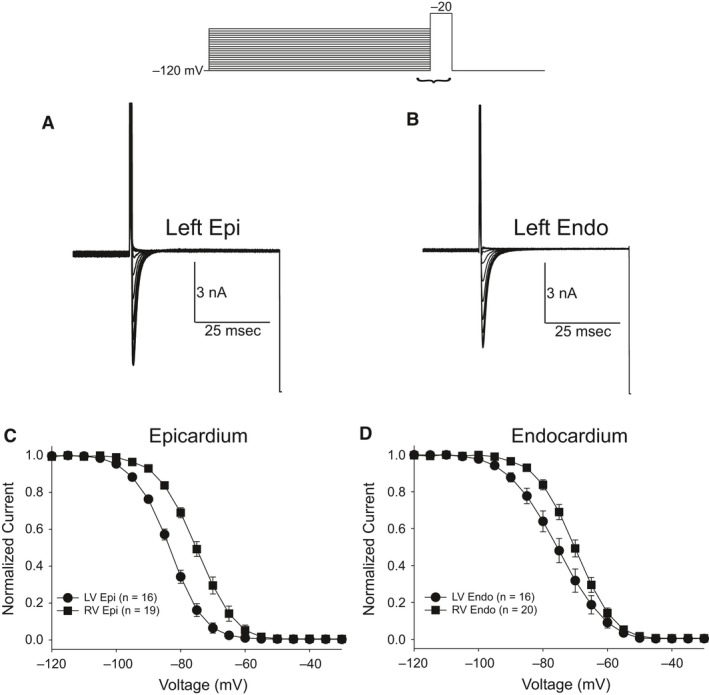
Representative steady‐state inactivation recordings from a LV epi (A) and LV endo myocyte (B). At the top of figure is the voltage clamp protocol. Peak current was normalized to their respective maximum values and plotted against the conditioning potential. The mean data for the steady‐state inactivation relation for epicardium (C) and endocardium (D) are shown.

We next evaluated steady‐state inactivation in LV and RV cells. After application of 500 msec prepulses, a test pulse to −20 mV was applied and peak current was normalized to the maximum current and plotted as a function of the prepulse voltage. A Boltzmann function was then fit to the data. Representative traces recorded from LV epi (Fig. 4A) and LV endo cells (Fig. 4B) are shown. Figure 4C shows there was a small but significant difference in the mid‐inactivation potential between LV and RV epi cells (*V*_1/2_ = −83.6 ± 0.07 mV, *k* = 5.36 ± 0.06 for LV epi vs. *V*_1/2_ = −75.3 ± 0.08 mV, *k* = 5.76 ± 0.07 for RV epi, *P* < 0.05). Similarly, Figure 4D shows there was a small but significant difference in the mid‐inactivation potential between LV and RV endo cells (*V*_1/2_ = −75.7 ± 0.13 mV, *k* = 7.07 ± 0.11 for LV endo vs. *V*_1/2_ = −70.3 ± 0.08 mV, *k* = 5.77 ± 0.07 for RV endo, *P* < 0.05).

**Figure 5:**
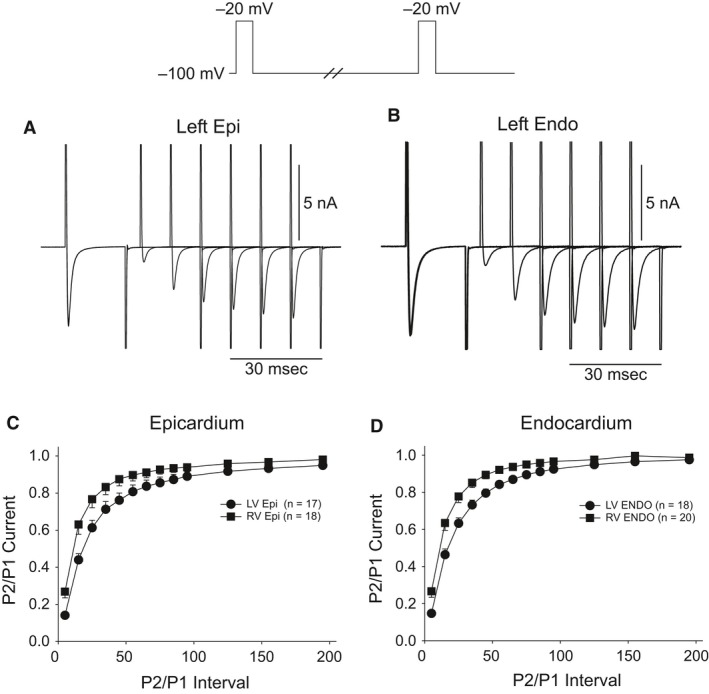
Representative traces recorded from a LV epi (A) and LV endo myocyte (B) showing recovery from inactivation. Recover was measured using two identical voltage clamp steps to −20 mV from a holding potential of −100 mV separated by varying time intervals. The mean data for recovery from inactivation for epicardium (C) and endocardium (D) are shown.

In the next series of experiments, we determined if recovery from inactivation of *I*_Na_ was different in the four cell types. Recovery was determined using a standard double‐pulse protocol separated by various time intervals (voltage clamp protocol shown at top of figure). Representative traces recorded from a LV epi and endo cell showing *I*_Na_ recovery at holding potential of −100 mV (Fig. 5A–B). Recovery of *I*_Na_ was slightly slower in LV cells compared to RV cells (Fig. 5C–D). In LV epi, reactivation of *I*_Na_ had a fast and slow phase of recovery as follows: (1) *τ*_1_ = 14.5 ± 0.85 msec and *τ*_2_ = 65.7 ± 9.7 msec. In contrast, RV epi cells exhibited a faster recovery from inactivation as follows: (1) *τ*_1_ = 9.5 ± 0.49 msec and *τ*_2_ = 51.9 ± 6.2 msec (*P* < 0.05 for fast and slow phase). In LV endo, reactivation of *I*_Na_ had a fast and slow phase of recovery: (1) *τ*_1_ = 12.8 ± 0.57 msec and *τ*_2_ = 45.6 ± 2.5 msec. Similarly, RV endo cells exhibited a faster recovery from inactivation as follows: (1) *τ*_1_ = 8.9 ± 0.81 msec and *τ*_2_ = 36.9 ± 4.7 msec (*P* < 0.05 for fast and slow phase).

**Figure 6:**
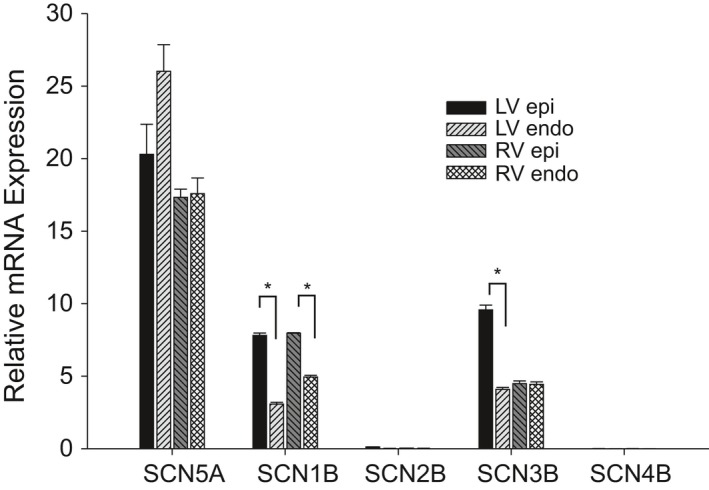
Bar graph comparing fold changes in left ventricle and right ventricular tissue for mRNA encoding five voltage‐gated sodium channel subunits in the canine heart. Expression was normalized from ∆*C*_t_ values for each gene against reference gene 18S. * denotes *P* < 0.05.

**Figure 7:**
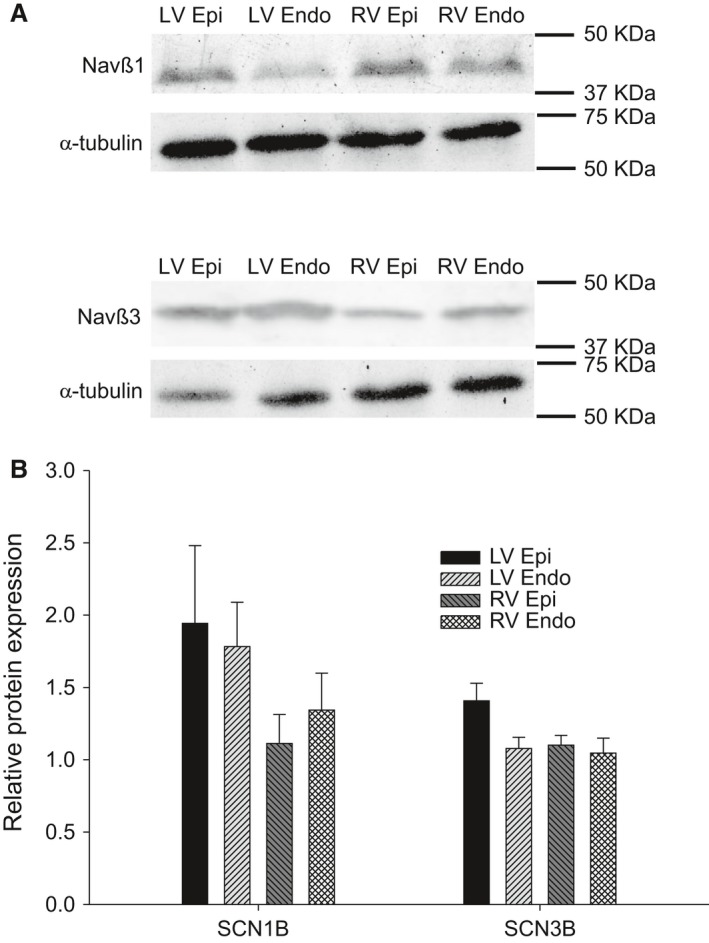
Representative Western blots and relative protein level (to *α*‐tubulin) of Na_v_ *β*1 and Na_v_ *β*3 in RV and LV (A). Mean data showing protein levels of Na_v_ *β*1 and Na_v_ *β*3. Tissue obtained from *n* = 3 animals.

Our biophysical results show a lower density of *I*_Na_ in RV epi and endo cells compared to LV epi and endo cells. In addition, other biophysical differences in the biophysical properties between RV and LV cells such as *V*_1/2_ of steady‐state inactivation and different time courses of recovery from inactivation were noted. These observations may suggest differences in the levels of the main alpha subunit Na_v_1.5 or the primary beta subunits (Nav*β*1‐*β*4). We next examined mRNA expression levels of *SCN5A* (encoding Na_v_1.5) and *SCN1B‐4B* (encoding *β*1‐4 subunit isoforms) in tissue isolated from the LV and RV. The expression for each Na^+^ channel subunit was compared to the housekeeping gene (18S) using quantitative RT‐PCR analysis (Fig. 6). We found that the alpha subunit, *SCN5A*, was the highest gene expressed compared with the beta subunits in the canine heart. These results show no difference in expression of *SCN5A* in the LV versus the RV. Results obtained for the beta subunits showed no significant difference in mRNA between RV and LV. However, epi cells expressed more Nav*β*1 compared to endo cells. Western blot analysis showed Nav*β*1 and Nav*β*3 were expressed at high levels in both ventricle but no significant differences were noted in the ventricular chambers (Fig. 7). mRNA levels of Nav*β*2 and *β*4 were expressed at very low levels compared to Nav*β*1 and *β*3 and analysis of Nav*β*2 and Nav*β*4 revealed that the protein appears to be absent in both LV and RV tissue (data not shown), in agreement with RT‐PCR experiments.

## Discussion

Our results show important biophysical and molecular differences in peak *I*_Na_. The LV wall is thicker than the RV wall, but transmural conduction time was similar between the two chambers, suggesting less *I*_Na_ in the RV. Voltage clamp analysis confirmed this observation as there was a lower density of peak *I*_Na_ in both RV epi and endo cells compared to LV epi and endo cells. Measurements of other biophysical properties showed that LV epi and endo cells had a more negative steady‐state inactivation *V*½ and slower recovery from inactivation compared to RV epi and endo cells. Examination of the sodium channel subunits showed no significant LV versus RV differences in the levels of SCN5A or SCN1B‐SCN4B expression. Interestingly, SCN2B and SCN4B expression was virtually absent in both LV and RV tissue. The slower conduction time in RV is likely attributable to the lower *I*_Na_ density in RV versus LV. Our results may partially explain why mutations in *SCN5A* (resulting in lower peak *I*_Na_) tend to predominantly affect the RV resulting in the Brugada phenotype.

Previous studies have observed regional variations in the biophysical properties of *I*_Na_. For example, studies have demonstrated a larger *I*_Na_ density in atria compared to ventricle (Li et al. 2002; Burashnikov et al. 2007; Calloe et al. 2011). Within the dog ventricle, we found that ventricular LV epi and LV endo have equivalent *I*_Na_ densities, but epi *I*_Na_ had a more negative steady‐state mid‐inactivation potential compared to endo (Cordeiro et al. 2008). In contrast, an epi‐endo gradient of *I*_Na_ was measured in mice (Veerman et al. 2017).

### Biophysical and molecular analysis of *I*_Na_

Differences in the biophysical properties of RV versus LV sodium channel manifestation, such as *I*_Na_ kinetics and magnitude, suggest differences in the makeup of the corresponding channel complex. It is known that *I*_Na_ in cardiac myocytes primarily reflects the activity of the Na_v_1.5 isoform (Maier et al. 2002; Haufe et al. 2005), but it is not wholly defined by the pore‐forming Na_v_1.5 *α*‐subunit. Other ‘auxiliary’ proteins, including several *β*‐subunits, are also involved. Interestingly, epi cells for either chamber expressed higher levels of Na_v_ *β*1 message compared to endo cells from the same chamber (Fig. 6), however, at protein level the difference between epi and endo was not significant, and there was a general tendency toward more protein in LV compared to RV. The expression of Na_v_ *β*3 subunits was similar in LV versus RV cells and we could not detect Na_v_ *β*2 or Na_v_ *β*4 at either the message or protein level suggesting these particular subunits are not present in dog ventricle. Studies have shown that the presence of Na_v_ *β* subunits can facilitate trafficking of Na_v_1.5 *α* channel subunit to the membrane, shift the *I*_Na_ steady‐state inactivation curve, and alter the rate of *I*_Na_ recovery from inactivation (Isom 2001). A recent study showed that the molecular mechanism responsible for differences in *I*_Na_ in atria versus ventricular rat cells could be attributed to lower Na_v_ *β*2 and Na_v_ *β*4 expression in atria compared to that in ventricle (Chen et al. 2016). The authors speculate the lower expression of these beta subunits is responsible for the distinct biophysical properties of *I*_Na_ in atria, namely, a more negative steady‐state inactivation, and a faster activation and inactivation. The authors further demonstrate a differential sensitivity of *I*_Na_ to the Na^+^ channel blocker dronedarone (Chen et al. 2016). However, as Na_v_ *β*2 and Na_v_ *β*4 expression is essentially absent from canine ventricular cells, these subunits are unlikely to contribute to the RV versus LV differences in canine *I*_Na_ we demonstrate in this study.

Besides *β*‐subunits, other ‘auxiliary’ proteins and interacting proteins such as Ankyrin‐B impart differential biophysical properties to cardiac *I*_Na_ depending on their specific constituency (Isom et al. 1994; Mohler et al. 2004; Boukens et al. 2013). Interestingly there are numerous studies highlighting the interplay between *I*_Na_ and *I*_to_. For example, HEY2 was found to modulate the amplitude of both *I*_to_ and *I*_Na_ in mice and was shown to be involved in the epi‐endo gradient of both currents (Veerman et al. 2017). In another study, the *I*_to_ *β*‐subunit KChIP2 was involved in regulating *I*_Na_ density (Nassal et al. 2016) in guinea pigs (a species that lacks Kv4.3 and *I*_to_). These studies further confirm that the biophysical properties of I_Na_ are not solely due to Na_v_1.5 and Na_v_ *β*1‐ Na_v_ *β*4.

### Differences under pathophysiological conditions

To our knowledge this is the first study examining *I*_Na_ density between LV and RV cells. Our results show there are significant differences in the inactivation characteristics, recovery, and density of Na^+^ channels in the two chambers. Consistent with our observations, a recent study demonstrated that acute sodium current inhibition in mouse hearts (with tetrodotoxin) resulted in frequency‐dependent conduction velocity slowing in the RV only, with the LV unaffected. The authors suggested that normal structural heterogeneities present in the RV are responsible for increased vulnerability to conduction slowing in the presence of reduced sodium channel function (Kelly et al. 2018). Alternatively, other studies have shown lower Na^+^ channel expression in the RVOT compared to the RV and have implicated the RVOT as the main origin of arrhythmias in BrS (Boukens et al. 2013). No matter the mechanism, this reduction in *I*_Na_ in the right ventricle may become important when other factors, such as *SCN5A* mutations, result in a loss of function. These *SCN5A* mutations would likely reduce *I*_Na_ in both chambers; however, it is clear that RV has less of a “depolarization” reserve than LV. Interestingly, *I*_to_ is larger in right ventricular epi compared to the left (Di Diego et al. 1996), resulting in a more prominent phase 1 repolarization compared to LV. This would lead to a greater depression of RV tissue, as the balance between inward and outward currents already favors the repolarizing forces and leads to conditions favorable for the development of the BrS phenotype due to loss of the dome (Calloe et al. 2009). The lower *I*_Na_ density together with the higher *I*_to_ density in the RV may explain why BrS mainly manifests in the RV (Brugada and Brugada 1992; Di Diego et al. 2002; Calloe et al. 2009).

### Clinical implications

BrS has been linked to decreased inward currents or increased outward currents during phase 1, accentuating the spike‐and‐dome morphology of the AP mainly in the epicardium (Calloe et al. 2009; Cordeiro et al. 2009). Two opposing hypothesis have been put forward to describe these observations (for review, see Wilde et al. (2010)). Our results would indicate that both the repolarization and the depolarization hypothesis can play a role in the development of BrS. Furthermore, it is clear that there is a differential expression of *I*_Na_ and *I*_to_ as well as other currents in different regions of the heart (Clark et al. 1993; Li et al. 2002; Calloe et al. 2011). It is likely that there are deviations in the levels of expression of the different currents (e.g., *I*_to_ vs. *I*_Na_) in different individuals, which may explain why the same mutation can manifest in different phenotypes, even within the same family.

## Conflicts of Interests

None to declare.
